# Curcumin elevates sirtuin level but does not postpone *in vitro* senescence of human cells building the vasculature

**DOI:** 10.18632/oncotarget.8450

**Published:** 2016-03-28

**Authors:** Wioleta Grabowska, Małgorzata Suszek, Maciej Wnuk, Anna Lewinska, Emilia Wasiak, Ewa Sikora, Anna Bielak-Zmijewska

**Affiliations:** ^1^ Department of Biochemistry, Nencki Institute of Experimental Biology, Polish Academy of Sciences, Warsaw, Poland; ^2^ Department of Genetics, University of Rzeszow, Rzeszów, Poland; ^3^ Department of Biochemistry and Cell Biology, University of Rzeszow, Rzeszów, Poland

**Keywords:** curcumin, senescence, sirtuins, VSMC, EC, Gerotarget

## Abstract

It is believed that curcumin, a component of the turmeric that belongs to hormetins, possesses anti-aging propensity. This property of curcumin can be partially explained by its influence on the level of sirtuins. Previously, we have shown that relatively high (2.5-10 μM) doses of curcumin induce senescence of cancer cells and cells building the vasculature. In the present study we examined whether curcumin at low doses (0.1 and 1 μM) is able to delay cell senescence and upregulate the level of sirtuins in human cells building the vasculature, namely vascular smooth muscle (VSMC) and endothelial (EC) cells. To this end we used cells senescing in a replicative and premature manner. We showed that low doses of curcumin in case of VSMC neither postponed the replicative senescence nor protected from premature senescence induced by doxorubicin. Moreover, curcumin slightly accelerated replicative senescence of EC. Despite some fluctuations, a clear increasing tendency in the level of sirtuins was observed in curcumin-treated young, senescing or already senescent cells. Sirtuin activation could be caused by the activation of AMPK resulting from superoxide elevation and ATP reduction. Our results show that curcumin at low doses can increase the level of sirtuins without delaying senescence of VSMC.

## INTRODUCTION

Curcumin, a natural compound derived from *Curcuma longa*, is considered as a potent anti-aging factor [1, 2]. There is a lot of data concerning its beneficial activity for the whole organism, including elongation of the life of model organisms. Curcumin is able to reduce the negative influence of some factors and proved beneficial in alleviating the symptoms of some diseases [3, 4, 5]. The most important activity of curcumin stems from its anti-inflammatory properties but there are also data suggesting the role of curcumin in sirtuin stimulation [6, 7, 8]. Sirtuins, NAD-dependent deacetylases, are involved in DNA repair, genome stability, telomere structure maintenance but also in epigenetic modifications of histones [9]. Their activity is considered as potentially anti-aging, therefore activators of sirtuins could be regarded as potential anti-aging compounds. It is believed that sirtuins are responsible for lifespan elongation of model organisms and are the key elements elevated during caloric restriction [10, 11]. They are also necessary for the proper functioning of the cardiovascular system [12, 13, 14]. With age, the level of sirtuin 1 and 6 decreases [15]. The lack of sirtuin 6 caused premature aging [16] and of sirtuin 1 promoted expression of genes, which are normally expressed during aging [17]. Sirtuin 1 prevented replicative senescence of normal human umbilical cord fibroblasts and antagonized both replicative and premature senescence in stem cells and differentiated cells under conditions of oxidative stress [18, 19]. Also sirtuin 3 is strongly correlated with the aging process and there are data which link this protein with longevity [20, 21, 22]. Although there are suggestions that curcumin acts by sirtuin activation [6, 7] such role in the process of cellular senescence is still not clear. It has been shown that curcumin can elongate the lifespan of *Caenorhabditis elegans* but not when the sirt2 gene (homolog of mammalian sirtuin 1) is mutated [3]. Moreover, pretreatment with curcumin attenuates mitochondrial oxidative damage induced by myocardial ischemia reperfusion injury by sirtuin 1 activation [7].

It has been suggested that curcumin is a hormetin, molecule which acts in a biphasic dose response manner [23]. In this study we explore the hypothesis that curcumin at low doses (0.1-1 μM) is able to postpone cellular senescence (replicative and premature) and to upregulate the level of sirtuins in cells building the vasculature, namely, human vascular smooth muscle and endothelial cells (VSMC and EC, respectively). Our results document that curcumin at low doses upregulated the level of sirtuins without delaying the senescence of cells building the vasculature.

## RESULTS

### Curcumin does not postpone replicative senescence of VSMC and EC

**Figure 1:**
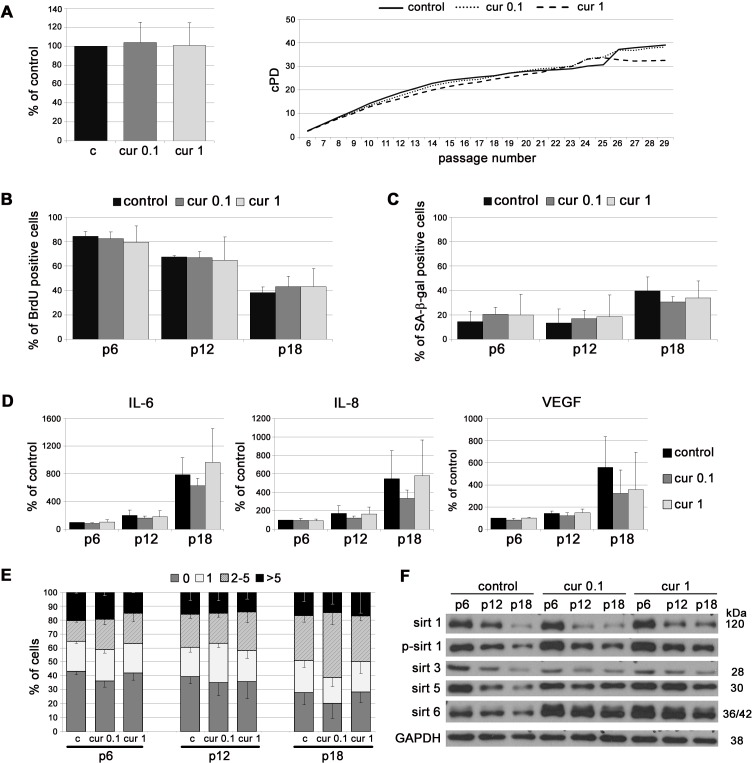
The impact of curcumin on replicative senescence of VSMC **A.** cPD of VSMC treated with different concentrations of curcumin (0.1 or 1 μM). Graphs show cPD of the last measured passage (left) and the average growth curves (right). **B.** Estimation of the proliferation rate by measurement of DNA synthesis as BrdU incorporation in VSMC cultured in medium supplemented with curcumin (0.1 or 1 μM) and investigated at passage 6, 12 and 18. The percentage of BrdU positive cells is presented on the graph. **C.** SA-β-gal activity in VSMC cultured in medium supplemented with curcumin (0.1 or 1 μM) and analyzed at passage 6, 12 and 18. The graph demonstrates the percentage of SA-β-gal-positive cells. **D.** Secretory phenotype (SASP) of VSMC cultured in medium supplemented with curcumin (0.1 or 1 μM) and investigated at passage 6, 12 and 18. The level of IL-6, IL-8 and VEGF is shown. **E.** DNA damage in VSMC cultured in medium supplemented with curcumin (0.1 or 1 μM) and analyzed at passage 6, 12 and 18. 0 - cells without DNA damage, 1 - with only one 53BP1 focus, 2-5 - with the number of foci between 2 and 5, > 5 - cells with more than five foci. **F.** Western blot analysis of sirtuin 1, 3, 5 and 6 level and phosphorylation of sirtuin 1 in VSMC cultured in medium supplemented with curcumin (0.1 or 1 μM) and collected at passage 6, 12 and 18. GAPDH served as a loading control. p - passage number, c - control, cur 0.1, cur 1 - 0.1 or 1 μM curcumin. Error bars indicate SD, *n* = 3 or more.

To analyze the impact of curcumin on replicative senescence *in vitro*, cells were cultured in medium supplemented with curcumin at concentrations that do not limit cell proliferation. The concentrations have been chosen experimentally and were 0.1 and 1 μM for VSMC, and 0.1 μM for EC [24]. VSMC, growing in medium supplemented with curcumin, proliferated similarly to control cells (cPD, BrdU incorporation). At late passages, in 1 μM curcumin, cPD was slightly lower than for control cells but without statistical significance (Figure 1A, 1B). The number of senescent cells, assessed as the number of senescence associated β galactosidase (SA-β-gal)-positive cells, was slightly lower in the population of curcumin-treated cells than in controls but without statistical significance (Figure 1C). At passage 18, curcumin (0.1 μM) slightly decreased the production of the mediators of inflammation, such as IL-6 and IL-8, as well as VEGF, but the differences were not statistically significant (Figure 1D). Analysis of DNA damage during replicative senescence, measured as a number of 53BP1 foci, revealed no effect of curcumin (Figure 1E). In VSMC, curcumin increased the level of sirtuin 1 and 6 at early passages. In particular, cells cultured with 1 μM curcumin had a higher level of these proteins and phosphorylated form of sirtuin 1 than control cells. The level of mitochondrial sirtuin 3 did not differ in comparison to control but the level of sirtuin 5 was elevated in curcumin treated cells (Figure 1F).

**Figure 2:**
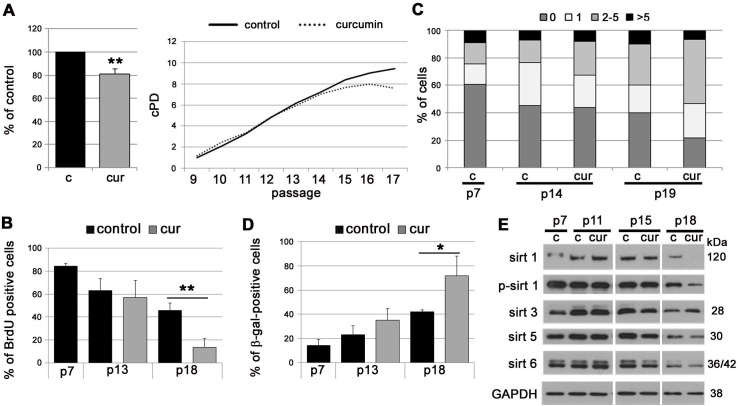
The impact of curcumin on replicative senescence of EC **A.** cPD of EC treated with curcumin (0.1 μM). Graphs show the cPD of the last measured passage, p18 (left) and the average growth curve (right). **B.** Estimation of the proliferation rate by measurement of DNA synthesis as BrdU incorporation in EC cultured in medium supplemented with curcumin (0.1 μM) and collected at passage 7, 13 and 18. The percentage of BrdU positive cells is presented on the graph. **C.** DNA damage in EC cultured in medium supplemented with curcumin (0.1 μM) and collected at passage 7, 14 and 19. 0 - cells without DNA damage, 1 - with only one 53BP1 focus, 2-5 - with the number of foci between 2 and 5, > 5 - cells with more than five foci. **D.** SA-β-gal activity in EC cultured in medium supplemented with curcumin (0.1 μM) and collected at passage 7, 13 and 18. The graph with the percentage of SA-β-gal-positive cells is shown. **E.** Western blot analysis of sirtuin 1, 3, 5 and 6 level and phosphorylation of sirtuin 1 in EC cultured in medium supplemented with curcumin (0.1 μM) and collected at passage 7, 11, 15 and 18. GAPDH served as a loading control. p - passage number, c - control, cur - 0.1 μM curcumin. Error bars indicate SD, *n* = 3 or more. * *p* < 0.05, ** *p* < 0.01, *** *p* < 0.001.

In EC, curcumin slightly accelerated replicative senescence. At first, cells proliferated similarly to untreated cells but since passage 14 they started to divide slower and stopped proliferating earlier than control cells (cPD, BrdU incorporation) (Figure 2A, 2B). Analysis of DNA double strand breaks (DSB) by visualization of the 53BP1 protein revealed that cells cultured in medium supplemented with curcumin, in comparison to controls, exhibited a higher level of DNA damage, quantified both as a number of DSB foci and as a number of cells with damaged DNA (Figure 2C). Curcumin increased the number of cells with elevated activity of SA-β-gal (Figure 2D) and decreased the level of all sirtuins (except sirtuin 3) during replicative senescence of EC (Figure 2E).

### Curcumin does not prevent premature senescence of VSMC induced by doxorubicin

**Figure 3:**
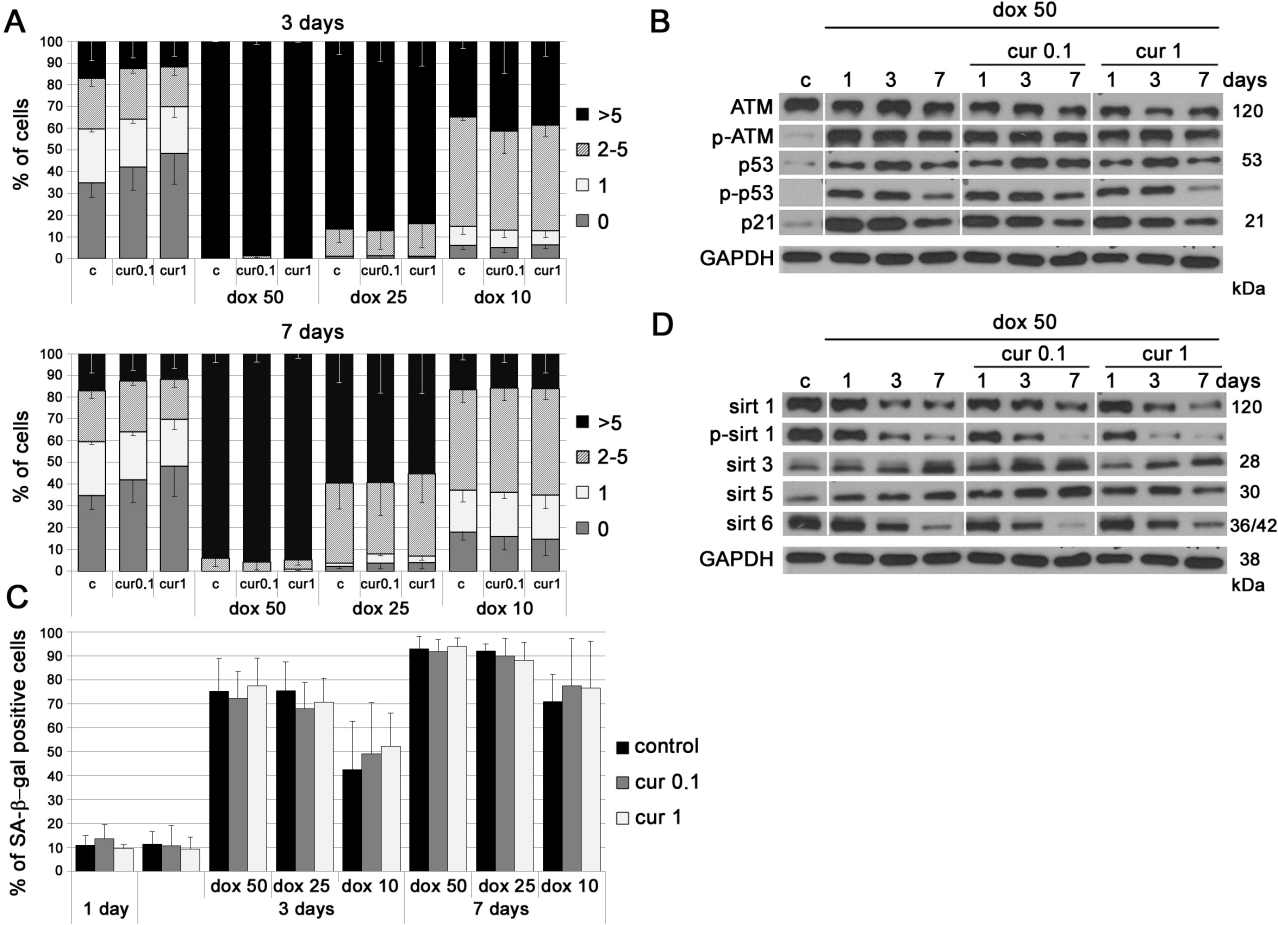
The impact of curcumin on stress induced premature senescence of VSMC **A.** Analysis of DNA damage in VSMC upon treatment with curcumin (0.1 or 1 μM) together with doxorubicin (10, 25 or 50 nM) expressed as a number of 53BP1 foci, after 3 and 7 days. 0 - cells without DNA damage, 1 - with only one focus, 2-5 - with the number of foci between 2 and 5, > 5 - cells with more than five foci. **B.** Western blot analysis of ATM, p53 total and phosphorylated, and p21 level in VSMC treated with doxorubicin (50 nM) and doxorubicin together with curcumin (0.1 or 1 μM). GAPDH served as a loading control. **C.** SA-β-gal activity in cells treated with curcumin (0.1 or 1 μM) or curcumin together with doxorubicin (10, 20 or 50 nM). The graph with the percentage of SA-β-gal-positive cells is shown. **D.** Western blot analysis of sirtuin 1, 3, 5 and 6 level and phosphorylation of sirtuin 1 after treatment with doxorubicin (50 nM) and doxorubicin together with curcumin (0.1 or 1 μM). GAPDH served as a loading control. All treated cells were in the phase of intensive growth (proliferating, young, passage 7-9). c - control; cur 0.1, cur 1 - 0.1 or 1 μM curcumin; dox 10, 25, 50 - 10, 25 or 50 nM doxorubicin. Error bars indicate SD, *n* = 3 or more.

We have shown earlier that curcumin in cytostatic concentrations induced cellular senescence even though it was able to reduce the number of DNA damage foci (less DNA DSB than in control cells) [24]. In this work we attempted to investigate whether curcumin in lower concentrations could protect cells from DNA damage induced by doxorubicin. We treated cells with doxorubicin together with curcumin and analyzed the level of DNA DSB after 3 and 7 days (Figure 3A). We used different concentrations of both curcumin (0.1 and 1 μM) and doxorubicin (10, 25 and 50 nM). Our results revealed that curcumin did not protect cells from DNA damage induced by doxorubicin as demonstrated by the analysis of the number of foci of the 53BP1 protein. Likewise, no spectacular changes were observed in the level of proteins involved in the DDR pathway and senescence, namely ATM, p53 and p21 (Figure 3B). Curcumin was not able to reduce the number of SA-β-gal-positive cells after 25 and 50 nM doxorubicin co-treatment either after 3 or 7 days of culture (Figure 3C). Surprisingly, treatment with the lowest concentration of doxorubicin, 10 nM, together with curcumin tended to increase the number of senescent cells as compared to VSMC treated with doxorubicin alone (statistically irrelevant). This suggested an additive effect of curcumin and doxorubicin under the applied experimental conditions. All studied sirtuins were downregulated (except sirtuin 3) and the levels were similar in cells treated with doxorubicin only (50 nM) or with doxorubicin and curcumin together (Figure 3D).

### Single application of low doses of curcumin (not inhibiting cell proliferation) decreases the level of the mediators of inflammation and increases the level of sirtuins

**Figure 4:**
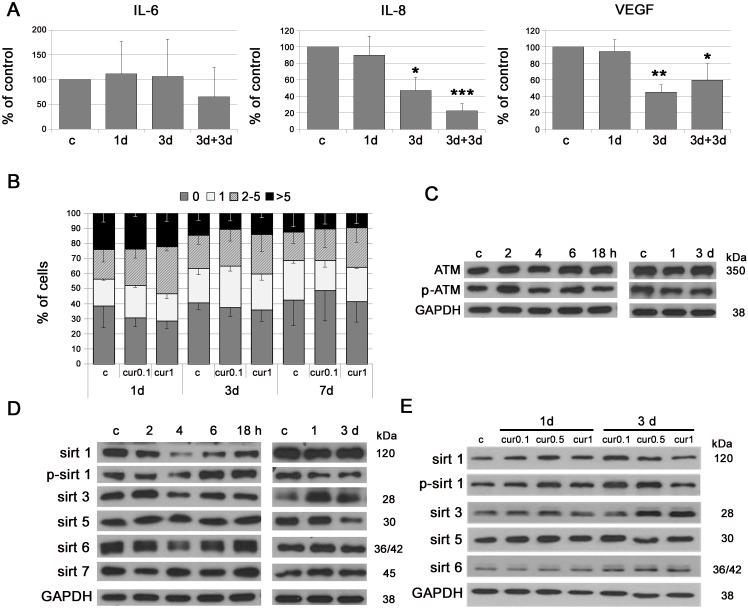
The impact of single application of low doses of curcumin on some parameters characteristic for cellular senescence **A.** Secretory phenotype (SASP) of VSMC treated with curcumin (0.1 μM). The level of IL-6, IL-8 and VEGF is shown. 3+3d - 3 days of curcumin treatment followed by 3 days of repeated curcumin supplementation. **B.** DNA damage in VSMC treated with curcumin (0.1 or 1 μM) during 1, 3 or 7 days expressed as the number of DNA DSBs visualized by 53BP1 immunocytochemistry. 0 - cells without DNA damage, 1 - with only one 53BP1 focus, 2-5 - with the number of foci between 2 and 5, > 5 - cells with more than five foci. **C.** Western blot analysis of total and phosphorylated ATM in VSMC treated with curcumin (1 μM) after 2, 4, 6, 18 hours and 1 and 3 days. GAPDH served as a loading control. **D.** Western blot analysis of sirtuin 1, 3, 5, 6 and 7 level and phosphorylation of sirtuin 1 in VSMC after 1 μM curcumin treatment for 2, 4, 6, 18 hours or 1 and 3 days. GAPDH served as a loading control. **E.** Western blot analysis of sirtuin 1, 3, 5 and 6 level and phosphorylation of sirtuin 1 in EC after curcumin (0.1, 0.5 and 1 μM) treatment for 1 and 3 days. GAPDH served as a loading control. c - control; cur 0.1, cur 0.5, cur 1 - 0.1, 0.5 or 1 μM curcumin; 2, 4, 6, 18h - 2, 4, 6 or 18 hours with curcumin; 1, 3d - 1 or 3 days after curcumin treatment. Error bars indicate SD, *n* = 3 or more. * *p* < 0.05, ** *p* < 0.01, *** *p* < 0.001.

We studied the effect of a single application of a low dose of curcumin (0.1 or 1 μM) in VSMC. Curcumin (0.1 μM) decreased the level of the mediators of inflammation, namely IL-8 and VEGF (Figure 4A). In the case of IL-6, the decrease (without statistical significance) was preceded by transient upregulation. Low doses of curcumin were not effective in reducing DNA damage (0.1 or 1 μM) (Figure 4B) and did not influence the level of ATM (1 μM) (Figure 4C). The analysis of the level of sirtuins revealed that curcumin (1 μM), within short time period (2-18h), increased phosphorylation of sirtuin 1 and the level of sirtuin 6, following a temporal decrease in both sirtuins. During longer culture (1-3 days) curcumin increased sirtuin 3 level but did not change the level of other sirtuins (Figure 4D). A time and dose dependent increase in sirtuin 1, 3 and 6 was observed in EC (Figure 4E).

### Curcumin applied to VSMC at late passages does not reduce senescence symptoms and increases the level of sirtuins

**Figure 5:**
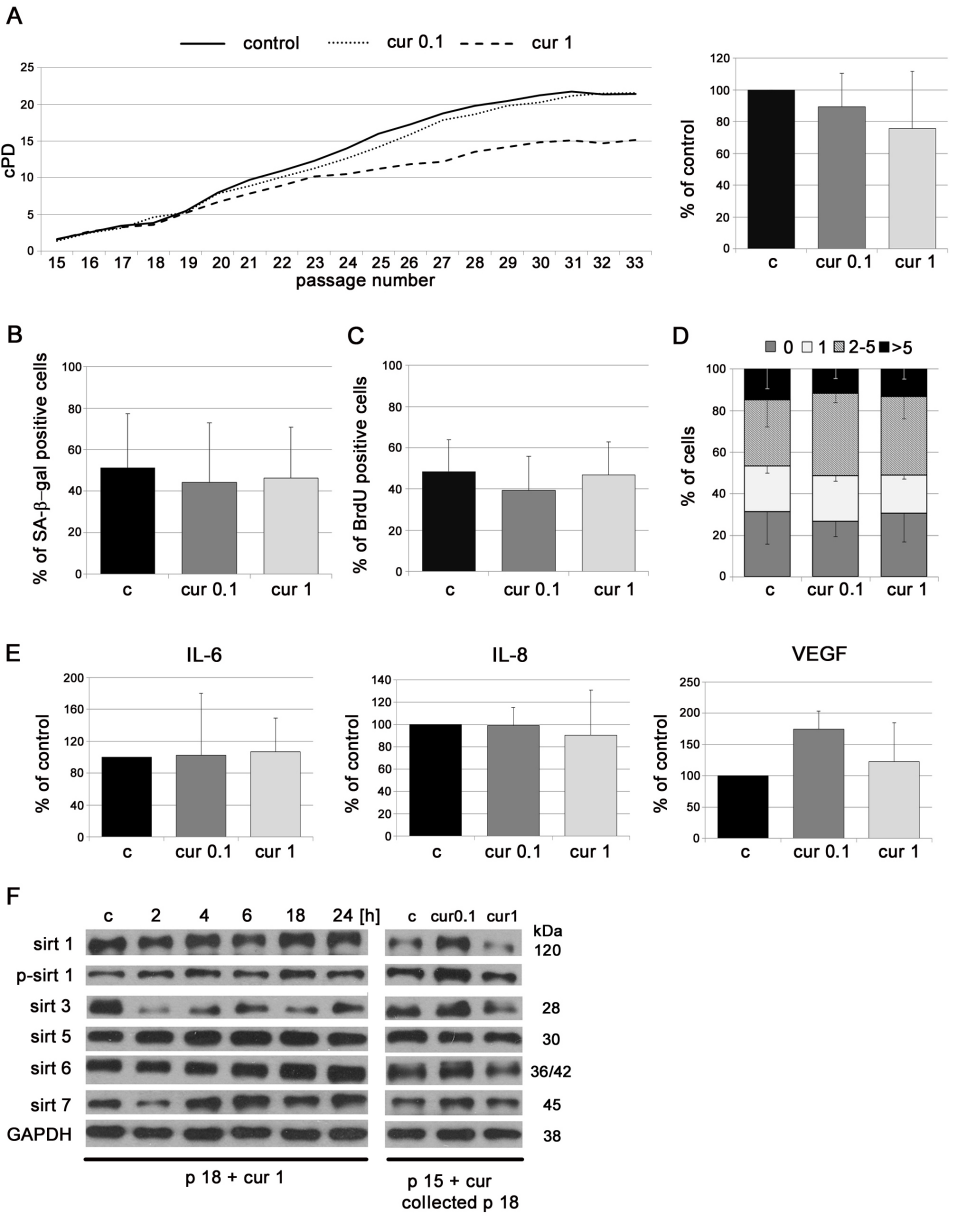
The impact of application of low doses of curcumin to VSMC at late passages on some parameters characteristic for cellular senescence **A.** cPD of VSMC treated with different concentrations of curcumin (0.1 or 1 μM). The average growth curve (left) and cPD at the last measured passage (right) are shown. **B.** SA-β-gal activity in VSMC cultured in medium supplemented with curcumin (0.1 or 1 μM) from passage 15 to 18, collected at passage 18. The graph demonstrates the percentage of SA-β-gal-positive cells. **C.** Estimation of the proliferation rate by measurement of DNA synthesis as BrdU incorporation in VSMC cultured in medium supplemented with curcumin (0.1 or 1 μM) from passage 15 to 18, collected at passage 18. The percentage of BrdU positive cells is presented on the graph. **D.** DNA damage in VSMC treated with curcumin (0.1 or 1 μM) from passage 15 to 18, collected at passage 18, expressed as the number of DNA DSBs visualized by 53BP1 immunocytochemistry. 0 - cells without DNA damage, 1 - with only one 53BP1 focus, 2-5 - with the number of foci between 2 and 5, > 5 - cells with more than five foci. **E.** Secretory phenotype (SASP) of VSMC cultured in medium supplemented with curcumin (0.1 or 1 μM) from passage 15 to 18, collected at passage 18. The level of IL-6, IL-8 and VEGF is shown. **F.** Western blot analysis of sirtuin 1, 3, 5, 6 and 7 level and phosphorylation of sirtuin 1 in VSMC after 1 μM curcumin treatment for 2, 4, 6, 18, 24 hours and treatment with 0,1 or 1 μM curcumin from passage 15 to 18. Cells treated for 2 - 24 hours were at passage 18 (p18). GAPDH served as a loading control. p - passage number, c - control; cur 0.1, cur 1 - 0.1, or 1μM curcumin; 2, 4, 6, 18 h - 2, 4, 6,18 or 24 hours with curcumin Error bars indicate SD, *n* = 3 or more.

As we have shown above, VSMC cultured constantly with curcumin did not senesce slower than control cells. We also wanted to examine whether curcumin acted differently when it was applied to cells at latter passages (p15). The analysis did not show that curcumin reduced the markers of senescence or the senescence rate in cells at the late passages. Cells treated with 0.1 μM curcumin proliferated similarly to untreated cells while higher concentration of curcumin, 1 μM, decreased the cPD (Figure 5A). No differences were observed in the rate of DNA synthesis (incorporation of BrdU) (Figure 5C), in the number of SA-β-gal-positive cells (Figure 5B) or in DNA damage assessed by visualization of 53BP1 foci (Figure 5D). Moreover, curcumin not only did not reduce the production of IL-6 and IL-8 but even increased the level of VEGF (Figure 5E). However, curcumin was able to increase the level of sirtuins. In senescent cells at passage 18, during the first 24h of curcumin treatment the level of sirtuin 1 (total and phosphorylated), sirtuins 5, 6 and 7 increased, while a spectacular reduction of sirtuin 3 was observed (Figure 5F). When cells were treated at passage 15 and cultured until passage 18, the effect was visible only in cells treated with 0.1 μM curcumin, in which the level of sirtuins 1, 3, 6, and 7 increased. At higher curcumin concentration (1 μM) the level of sirtuins 1, 6 and 7 did not differ in comparison to untreated cells or decreased as in the case of sirtuin 3.

### Curcumin increases the level of superoxide and AMPK, and decreases the level of ATP in VSMC which can be responsible for the activation and upregulation of sirtuins

**Figure 6:**
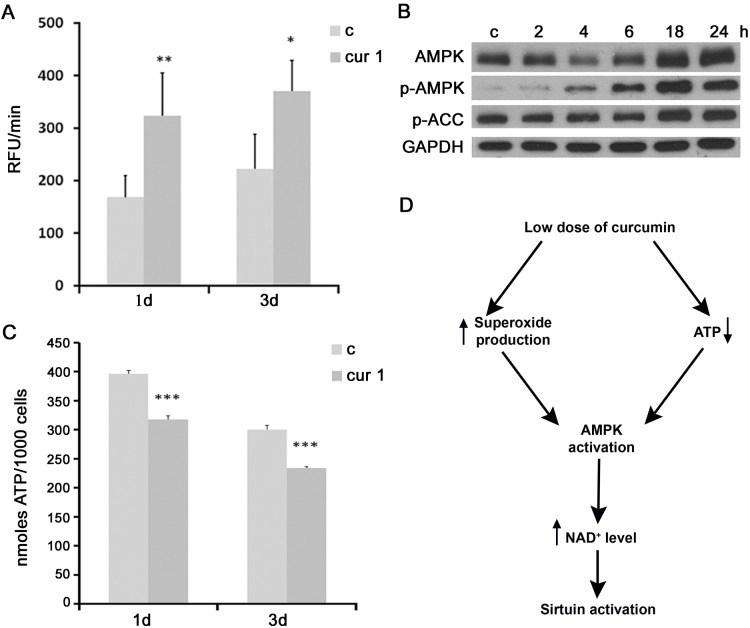
The impact of curcumin on superoxide production, AMPK activity and ATP level **A.** Analysis of superoxide production in VSMC cells treated with 1 μM curcumin for 1 or 3 days. **B.** Western blot analysis of AMPK level and phosphorylation and phosphorylation of its substrate ACC in VSMC after 1 μM curcumin treatment for 2, 4, 6, 18, 24 hours. Cells were at passage 7. GAPDH served as a loading control. **C.** ATP analysis in VSMC cells treated with 1 μM curcumin for 1 or 3 days. c - control; cur 1 - 1 μM curcumin; 2, 4, 6, 18, 24h - 2, 4, 6 18 or 24 hours with curcumin; 1, 3d - 1 or 3 days of curcumin treatment. Error bars indicate SD, *n* = 3 or more. * *p* < 0.05, **p < 0.01, *** *p* < 0.001. **D.** Proposed mechanism underlying curcumin-mediated sirtuin activation in VSMC cells.

It has been shown that a mild oxidative stress induced the expression of sirtuins (a compensatory mechanism), while acute or prolonged oxidative conditions caused sirtuin downregulation [25]. Curcumin, dependently on the circumstances, can act as pro- or antioxidant [26]. Our results demonstrated that curcumin in low doses increased the level of superoxide production (Figure 6A). Increased ROS production could be responsible for activation of AMPK (AMP-activated kinase) [27]. Indeed, our results revealed that cell treatment with curcumin (1 μM) increased the level and activity of AMPK, which is also supported by the increased phosphorylation of its substrate, ACC (Figure 6B). AMPK could be also induced by a decreased level of ATP, because it is an AMP-dependent protein. In this respect, we documented that curcumin reduced the ATP level (Figure 6C). AMPK is able to activate NAMPT, nicotinamide phosphoribosyltransferase, responsible for NAD^+^ synthesis. Sirtuins are NAD^+^-dependent and the increased ratio NAD^+^/NADP favors sirtuin activation. Therefore, one of the probable mechanisms by which curcumin could increase the activity of sirtuins is by inducing AMPK and, in consequence, increasing NAD^+^ synthesis. A proposed mechanism underlying curcumin-mediated sirtuin activation is presented in Figure 6D.

## DISCUSSION

The impact of curcumin is dose-dependent. Curcumin can act cytotoxically (above 7.5 μM, cell type dependently), cytostatically - pro-senescent activity (2.5-7.5 μM) [24], and it is believed that low doses (below 1 μM) have a beneficial effect for the organism, in accordance with the hormetical activity of this compound. We hypothesized that low doses of curcumin could potentially postpone cellular senescence. The bioavailability of curcumin is very low and after diet supplementation only about 1% of the dose was found in the serum which corresponds to nanomolar concentrations. The highest concentration observed in the blood was about 3.6 μM, but it was a result of extremely high consumption: 8 g per day for 3 months [28]. Curcumin concentrations used by us correspond to those detected in the serum. Surprisingly, we have shown that low doses of curcumin, which do not inhibit cell proliferation [24], did not delay the replicative senescence of VSMC and EC and did not protect VSMC from stress induced premature senescence. Moreover, replicative senescence of EC proceeded in an apparently accelerated manner. It suggested that EC were more sensitive to curcumin than VSMC. We speculate that the difference between VSMC and EC in the response to curcumin during replicative senescence could be the result of the ability of curcumin to upregulate the level of sirtuins 1 and 6, which seems to be cell type specific. In VSMC the level of sirtuin 1 and 6 increased after curcumin treatment and this protected cells from premature senescence (compensatory mechanism), while in EC curcumin did not influence the sirtuin level and therefore cells underwent senescence quicker than the control ones. Additionally, we observed that in VSMC curcumin reduced cell proliferation at late passages. We postulate that senescent cells are more sensitive to stress because the stress response machinery, due to decreasing activity of antioxidant enzymes with age [29], is less effective than in young cells. Therefore 1 μM curcumin did not reduce the proliferation potential of young cells but inhibited growth of presenescent cells.

Our previous research demonstrated the ability of curcumin to induce senescence, which can be considered as a type of anti-cancer action in the case of tumor cells [30] or as a side effect in the case of normal cells [24]. There is however only little information about the role of curcumin in the protection against cellular senescence. Curcumin improved the ability of normal human epidermal keratinocytes (at late passages) to differentiate during replicative senescence by induction of mild stress [26, 31]. In contrast to the limited knowledge about the anti-senescent function of curcumin, there is a plethora of data concerning such function of sirtuins (especially 1 and 6). The lack of sirtuin 6 led to premature aging and of sirtuin 1 caused expression of genes characteristic for aging [16, 17]. Moreover, sirtuin 1 delayed replicative senescence of normal human umbilical cord fibroblasts as well as replicative and premature senescence of stem cells and differentiated cells exposed to oxidative stress [18, 19]. Sirtuins antagonized cellular senescence of primary cells such as mouse embryonic fibroblasts, human diploid fibroblasts and human endothelial cells - including HUVEC [32 - 35, 14]. One of the possible mechanisms of curcumin activity is the upregulation of sirtuins. The protecting function of curcumin owed to the induction of sirtuins has been already exploited, for example, to reduce cisplatin chemotherapy-induced nephrotoxicity in rats [36]. We postulate that sirtuin activation can be involved in the protection from aging. As we have shown, in VSMC curcumin is able to upregulate the level of sirtuins in all tested experimental conditions. It is difficult to explain in which manner curcumin increases the level and activity of sirtuins (direct or indirect influence) because of its pleiotropic action. The possible mechanism could involve an increase of AMPK level and activity (Figure 6D). AMPK, an enzyme responsible for elevation of NAD^+^ level [37], can be induced by increased ROS production [27] as well as by decrease of ATP level. We have shown both the pro-oxidant activity of curcumin and the ability to reduce ATP level. Furthermore, AMPK is able to induce the transcription factor FOXO [38], which is involved in regulation of sirtuins expression.

Summarizing, we have shown that curcumin did not protect cells building the vasculature from senescence. However, it was able to upregulate the level of sirtuins 1, 3, 5, 6 and 7. Curcumin might activate/upregulate sirtuins by AMPK activation elicited by ROS elevation and ATP reduction. Our results suggest that the beneficial anti-aging effect attributed to curcumin is not caused by postponing cellular senescence and we propose that it could be due to the activation of sirtuins. However, even though curcumin did not postpone cellular senescence *in vitro,* it cannot be excluded that it may act differently *in vivo*. One of the activities of sirtuins is the epigenetic regulation of the activity of NFκB. This transcription factor, responsible for inflammation and associated with both cellular senescence and aging [39, 40], is the most recognized anti-inflammatory molecular target of curcumin. Curcumin inhibits NFκB *via* its effect on the kinase necessary to dissociate IκB (inhibitor of kappa B). Sirtuin 6 is able to deacetylate histone H3 at lysine 9 at the promoter of RELA (component of NFκB), causing inhibition of transcription and loss of NFκB activity [41]. Taking into account that curcumin increased the level of sirtuin 6, it can be assumed that there is another way of NFκB inhibition by curcumin. Moreover, it has been shown that other factors, including resveratrol, cilostazol, paeonol, statins and hydrogen sulfide, could protect cells from senescence by regulating sirtuin 1 [42 - 46]. Activators of sirtuins are considered as potential anti-aging and CVD protecting factors [47]. Sirtuin 1 is responsible for the beneficial effect of mild physical activity [48, 49] and supplementation of the diet with curcumin improved the effects of physical activity [50]. On the other hand, downregulation of sirtuins caused premature senescence [51]. Furthermore, sirtuins are considered as markers of frailty, which is one of the most important features of aging [52]. As it has been proposed by Rattan and Ali [53], repeated mild heat stress (RMHS) could act as an anti-aging factor. The authors suggested that curcumin, as a hormetin, could support the effect of RMHS and such combination could lead to the delay of aging. Activation of sirtuins was observed during cell response to a mild stress [25]. Thus mild stress leads to beneficial effects due to activation of stress response, conditioning and finally protection, including anti-aging [54]. We postulate that curcumin could act by itself as a mild stressor and in this manner cause beneficial effects for the organism.

## MATERIALS AND METHODS

### Reagents

Curcumin (C1386) was from Cayman (Ann Arbor, USA); dimethyl sulfoxide (DMSO) (D4540), doxorubicin and DAPI were purchased from Sigma-Aldrich (St. Louis, USA); BSA was from BioShop (Burlington, Canada).

### Culture of vascular smooth muscle cells (VSMC) and endothelial cells (EC)

Human primary VSMC and EC were purchased from ATCC or from Lonza. VSMCs were cultured in Vascular Cell Basal Medium (ATCC, LGC, Manassass, USA) supplemented as defined by the manufacturer. ECs were cultured in Vascular Cell Basal Medium (ATCC, LGC, Manassass, USA) supplemented as defined by the manufacturer. All cells were kept in humidified atmosphere (37°C and 5% CO_2_ in the air). The cells were passaged every 3-4 days and were seeded 24 hours before treatment at a density of 3-3,5×10^3^ cells/cm^2^ (VSMCs) and 10×10^3^ cells/cm^2^ (ECs). Cells at early passages (5-9) was described as young cells, at passages 10-14 as middle passage, at late passages (15-19) as senescent cells and cells at passages 20 or more as extremely senescent. Cell morphology was analyzed in an inverted light microscope Nikon. Curcumin was dissolved in DMSO and the final concentration of DMSO in cell culture did not exceed 0.1%. Doxorubicin was dissolved in the medium.

### cPD analysis

Cumulative population doubling (cPD) is a total population doubling (PD) which was calculated according to the formula:

$$\text{PD}=\frac{\log_{10}(N_{h})-\log_{10}(N_{i})}{\log_{10}(2)}$$

where: N_i_ (inoculum number) - the number of seeded cells, N_h_ (cell harvest number) - the number of collected cells in a defined period of time.

### Cell proliferation analysis

Cell proliferation was monitored by assessing the cell number in an improved Neubauer chamber. To assess DNA synthesis bromodeoxyuridine assay was used (BrdU, Sigma-Aldrich, St. Louis, USA). BrdU was added to the medium (10 μM) and cells were cultured for 24 h. Cells were washed in PBS, fixed with 70% EtOH and kept at least overnight at −20°C. BrdU was detected by using primary antibody against BrdU (Becton Dickinson, New Jersey, USA) and a secondary Alexa 488-conjugated antibody. DNA was stained with DAPI. Cells were analyzed under fluorescence Nikon Eclipse 50i microscope and the Image-Pro Plus 6.0 software. Cells were counted and the % of BrdU positive cells is shown on graphs.

### Quantification of senescence associated-β-galactosidase -positive cells

Detection of senescence associated-β-galactosidase (SA-β-gal) activity was performed according to Dimri et al. [55]. Briefly, cells were fixed with 2% formaldehyde, 0.2% glutaraldehyde in PBS, washed, and exposed overnight at 37°C to a solution containing 1 mg/ml 5-bromo-4-chloro-3-indolyl-b-D-galactopyranoside, 5 mM potassium ferrocyanide, 5 mM potassium ferricyanide, 150 mM NaCl, 2 mM MgCl_2_, and 0.1 M phosphate buffer, pH 6.0. Cells (100 or more) were counted under a light Nikon Eclipse 50i microscope and the % of SA-β-gal-positive cells was calculated.

### Measurements of secreted factors

Secretory phenotype was analyzed by ELISA in culture medium collected on days 1, 3 and 7 or at passages 6, 12 and 18. Experiments were conducted according to the protocol provided by the manufacturer (R&D Systems, Minneapolis, USA). Levels of cytokines (IL-6, IL-8, VEGF) in the samples were determined with the use of standard curves and normalized to cell number. Absorbance was measured at 450 nm using a Tecan Sunrise spectrophotometer (Tecan) and analyzed with the X-fluor 4 software.

### Western blotting analysis

Whole cell protein extracts were prepared according to Laemmli [56]. The primary antibodies used were: anti-ATM (1:500), anti-phospho-ATM Ser1981 (1:500) (Abcam, Cambridge, UK); anti-GAPDH (1:50000) (Millipore, Darmstadt, Germany); anti-p53 (1:500) (Santa Cruz Biotechnology, Santa Cruz, USA); anti-p21 (1:500) (Sigma-Aldrich, St. Louis, USA); anti-phospho-p53 Ser15 (1:250), anti-sirt 1 (1:250), anti-phospho-sirt 1 Ser47 (1:250), anti-sirt 3 (1:500), anti-sirt 5 (1:500), anti-sirt 6 (1:1000), anti-sirt 7 (1:250) anti-AMPKα (1:500), anti-phospho-AMPKα Thr172 (1:1000), anti-phospho-ACC Ser79 (1:1000) (Cell Signaling Technology, Denvers, USA). The respective proteins were detected after incubation with the horseradish peroxidase-conjugated secondary antibodies (1:2000) (Dako, Glostrup, Denmark), using an ECL system (Thermo Scientific, Rockford, USA, according to the manufacturer's instructions.

### Immunocytochemistry

For detection of 53BP1 foci cells grown on cover slides were washed and fixed with cold (−20°C) 70% ethanol at least overnight at −20°C. Next, cells were blocked with 5% bovine serum albumin (BSA) in PBS containing 0.5% Tween-20 and 0.1% Triton X-100 for 30 min. After washing the cells were incubated with primary anti-53BP1 antibody (Novus Biological, Cambridge, UK) diluted 1:500 in 1% BSA/PBS (0.5% Tween-20 and 0.1% Triton X-100) for 2h and with Alexa 488 secondary antibody (Life Technologies, Eugene, USA), 1:500 in 1% BSA/PBS (0,5% Tween-20 and 0.1% Triton X-100) for 1 h. DNA was stained with DAPI. 53BP1 foci were visualized under a fluorescence microscope.

### Measurement of superoxide production

Superoxide production was measured with dihydroethidium (Life Technologies, Eugene, USA). VSMC were washed and suspended in PBS containing 0.1% glucose, 0.5 mM EDTA and 5 μM dihydroethidium. After 15-min incubation in the dark at 37°C, fluorescence emitted due to oxidation of dihydroethidium to ethidium was monitored in a Tecan Infinite^®^ M200 fluorescence mode microplate reader. Measurement conditions were: λ_ex_ = 518 nm and λ_em_ = 605 nm; temperature 37°C. Data are presented as RFU (relative fluorescence unit) per minute.

### Measurement of ATP level

ATP concentration was measured using ViaLight™ Plus Cell Proliferation and Cytotoxicity BioAssay Kit (Lonza, Basel, Switzerland) according to the manufacturer's instructions. Briefly, VSMC were lysed with Cell Lysis Reagent (10 min, room temperature) and ATP Monitoring Reagent Plus (AMR Plus) was added to generate luminescent signal. After 2-min incubation, luminescence was monitored in a Tecan Infinite^®^ M200 luminescence mode microplate reader. Read time was 1 s (integrated). Calculation was made on the basis of a standard curve obtained for an ATP solution and intracellular ATP content was presented as nanomoles of ATP per 1 × 10^3^ cells.

### Statistical analysis

Statistical analysis was performed using 2-tailed Student /t/ test. Data are presented as a mean ±SD. A value of *p* < 0.05 was considered statistically significant (*p* < 0.05-*, *p* < 0.01-**, *p* < 0.001-***). All graphs show the mean results from at least 3 independent experiments.
